# Long-term co-circulation of multiple arboviruses in southeast Australia revealed by xeno-monitoring and viral whole-genome sequencing

**DOI:** 10.1093/ve/veae103

**Published:** 2024-11-25

**Authors:** Carla Julia S. P Vieira, Michael B Onn, Martin A Shivas, Damien Shearman, Jonathan M Darbro, Melissa Graham, Lucas Freitas, Andrew F van den Hurk, Francesca D Frentiu, Gabriel L Wallau, Gregor J Devine

**Affiliations:** Mosquito Control Laboratory, QIMR Berghofer Medical Research Institute, 300 Herston Road, Herston, QLD 4006, Australia; Centre for Immunology and Infection Control, School of Biomedical Sciences, Queensland University of Technology, 300 Herston Road, Herston, QLD 4006, Australia; Entomology Laboratory, Public Space Operations, Brisbane City Council, 20 Tradecoast Dr, Eagle Farm, QLD 4009, Australia; Entomology Laboratory, Public Space Operations, Brisbane City Council, 20 Tradecoast Dr, Eagle Farm, QLD 4009, Australia; Metro North Public Health Unit, Queensland Health, Briden Street, Windsor, QLD 4030, Australia; Metro North Public Health Unit, Queensland Health, Briden Street, Windsor, QLD 4030, Australia; Mosquito Control Laboratory, QIMR Berghofer Medical Research Institute, 300 Herston Road, Herston, QLD 4006, Australia; Australian Defence Force Malaria and Infectious Disease Institute, Gallipoli Barracks, Enoggera, QLD 4051, Australia; Global Data Science Initiative (GISAID) at, Oswaldo Cruz Foundation (FIOCRUZ), Avenida Brasil 4365, Rio de Janeiro, RJ 21040-360, Brazil; Department of Health, Public Health Virology, Forensic and Scientific Services, Queensland Government, 39 Kessels Road, Coopers Plains, QLD 4108, Australia; Centre for Immunology and Infection Control, School of Biomedical Sciences, Queensland University of Technology, 300 Herston Road, Herston, QLD 4006, Australia; Department of Entomology and Bioinformatic Core, Aggeu Magalhães Institute, Oswaldo Cruz Foundation (FIOCRUZ), Avenida Professor Moraes Rego, Recife, PE 50740-465, Brazil; Department of Arbovirology, Bernhard Nocht Institute for Tropical Medicine, WHO Collaborating Center for Arbovirus and Hemorrhagic Fever Reference and Research, National Reference Center for Tropical Infectious Diseases, Bernhard-Nocht-Street 74, Hamburg 20359, Germany; Mosquito Control Laboratory, QIMR Berghofer Medical Research Institute, 300 Herston Road, Herston, QLD 4006, Australia

**Keywords:** qRT-PCR, RNA sequencing, arbovirus surveillance, genomics

## Abstract

Arbovirus surveillance of wild-caught mosquitoes is an affordable and sensitive means of monitoring virus transmission dynamics at various spatial-temporal scales, and emergence and re-emergence during epidemic and interepidemic periods. A variety of molecular diagnostics for arbovirus screening of mosquitoes (known as xeno-monitoring) are available, but most provide limited information about virus diversity. Polymerase chain reaction (PCR)-based screening coupled with RNA sequencing is an increasingly affordable and sensitive pipeline for integrating complete viral genome sequencing into surveillance programs. This enables large-scale, high-throughput arbovirus screening from diverse samples. We collected mosquitoes in CO_2_-baited light traps from five urban parks in Brisbane from March 2021 to May 2022. Mosquito pools of ≤200 specimens were screened for alphaviruses and flaviviruses using virus genus-specific primers and reverse transcription quantitative PCR (qRT-PCR). A subset of virus-positive samples was then processed using a mosquito-specific ribosomal RNA depletion method and then sequenced on the Illumina NextSeq. Overall, 54,670 mosquitoes representing 26 species were screened in 382 pools. Thirty detections of arboviruses were made in 28 pools. Twenty of these positive pools were further characterized using RNA sequencing generating 18 full-length genomes. These full-length sequences belonged to four medically relevant arboviruses: Barmah Forest, Ross River, Sindbis-like, and Stratford viruses. Phylogenetic and evolutionary analyses revealed the evolutionary progression of arbovirus lineages over the last 100 years, demonstrating that different epidemiological, immunological, and evolutionary processes may actively shape the evolution of Australian arboviruses. These results underscore the need for more genomic surveillance data to explore the complex evolutionary pressures acting on arboviruses. Overall, our findings highlight the effectiveness of our methodology, which can be applied broadly to enhance arbovirus surveillance in various ecological contexts and improve understanding of transmission dynamics.

## 1. Introduction

In recent decades, arthropod-borne viruses (arboviruses) have emerged or re-emerged as important human and animal pathogens with tremendous implications for public health globally (Huang et al. 2019). Dengue viruses (DENV) alone infect around 390 million people annually (WHO 2023) and are associated with a substantial economic burden (US$40 billion) (Selck et al. 2014, Stanaway et al. 2016). Although many arboviruses were geographically restricted, the continued movement of people and vectors around the globe have expanded their ranges (Huang et al. 2019). For example, DENV and Chikungunya (CHIKV), both emerging in Africa, have now spread to every continent except Antarctica (Gubler 1998, Nunes et al. 2015) while Japanese encephalitis virus (JEV) has been expanding its range in Asia, and has recently become endemic across Australia (Yakob et al. 2023). Climate heating, extreme weather events, and anthropogenic factors such as land-use, urbanization, and globalization facilitate that invasion process by mediating changes in vector and reservoir distributions, and increasing immigration of viraemic hosts (Patz and Norris 2004, Vieira et al. 2020).

In Australia, endemic arboviruses cause a significant health and economic burden. They include the arthritogenic alphaviruses Ross River virus (RRV) and Barmah Forest virus (BFV), which respectively cause approximately 5000 and 1000 cases annually, and inducing significant health impacts (Australian Government 2024). These alphaviruses are also endemic in Papua New Guinea (PNG) (Kizu et al. 2023) while RRV has spread across several Pacific Island Countries and Territories (PICTs) where it caused an epidemic of an estimated 500,000 cases in 1979–80 (Marshall and Miles 1984). RRV has the potential for global spread (Lau et al. 2017, Shanks 2019). Additionally, Australia experiences sporadic outbreaks of encephalitogenic flaviviruses, including the Kunjin strain of West Nile virus (WNV_KUN_), Murray Valley encephalitis virus (MVEV), and JEV, with mortality rates of 15% for the 2022 JEV outbreak and 23% for the 2023 MVE outbreak (Australian Government 2024). Over 75 other alpha- and flaviviruses are endemic in Australia (CDC 2023), some with the potential to cause symptoms in humans. Their public health significance remains poorly understood as do their key vectors, reservoirs, and spillover risks (Gyawali et al. 2019).

Most arboviruses that infect humans belong to the *Alphavirus* and *Flavivirus* genera, in the Togaviridae and Flaviviridae families, respectively. The majority of human infections by these viruses are asymptomatic or subclinical meaning that the emergence or re-emergence of arboviruses may go undetected and unreported (Grubaugh et al. 2019b). A salient case is cryptic circulation of the Zika virus (ZIKV) in Brazil for more than 18 months before its first detection and facilitating its spread to over 40 countries (Faria et al. 2017, Grubaugh et al. 2019c). Another example in the Australian context is the recent JEV range expansion from Indonesia to Australia. The genotype of JEV currently circulating in Australia was detected in the Tiwi Islands, Northern Territory (NT), in February 2021, and 1 year later in other states of Australia (Sikazwe et al. 2022). However, it is estimated that the Australian clade emerged 6 years ago, suggesting that this clade of genotype IV (GIV) viruses had been cryptically circulating in Australia for several years [95% highest posterior density (HPD) 2–14] (Xu et al. 2023). There were 45 human cases of this vaccine-preventable disease recorded during 2022, including seven deaths. It also caused significant stock losses in piggeries (Zhang et al. 2023). It is possible that detrimental impacts of ZIKV in Brazil and JEV in Australia could have been reduced had there been an effective continuous surveillance for early detection and mitigation of these arboviruses, particularly with regard to vector control measures for *Aedes aegypti* (Ritchie et al. 2021) and the provision and targeting of JEV vaccines in humans (Furuya-kanamori et al. 2022).

Mosquito surveillance programs can play a pivotal role in public health strategy and disease preparedness. Xeno-monitoring—the detection of pathogens in arthropod vectors—is a widely used technique for (I) monitoring the distribution of existing and emerging pathogens (Cameron and Ramesh 2021) and (ii) characterizing the risks and pathways associated with their transmission to humans. Xeno-monitoring of arboviruses from field-collected mosquitoes can be conducted using various methods, including (i) virus culture, (II) PCR-based detection, e.g. quantitative real-time reverse transcription PCR (qRT-PCR), and, more recently, (III) sequencing, e.g. next-generation sequencing (NGS). These techniques have been extensively employed to map an enormous diversity of arboviruses across different mosquito populations and environments (Moonen et al. 2023). The NGS approaches, e.g. total RNA sequencing, facilitate the non-targeted, high-throughput detection and characterization of whole arbovirus genomes (Batovska et al. 2019, Pronyk et al. 2023). This powerful tool can assist in the characterization of novel or emerging pathogens or yield genome-wide information that increases our understanding of the transmission dynamics of different virus genomes in different environments and hosts (Batovska et al. 2018, Young et al. 2020).

In Australia, besides monitoring human cases, most states and territories conduct active arbovirus surveillance. This includes seasonally screening mosquitoes for specific arboviruses that are nationally notifiable, using virus isolation and/or qRT-PCR techniques (Knope et al. 2019). Sequencing is not a routine part of the surveillance, so, despite the wide distribution and clinical significance of several arboviruses, whole-genome sequences have only been recorded in a few studies (Michie et al. 2020, 2021, 2023). Although NGS is widely available in developed countries (Pronyk et al. 2023), genomic surveillance networks for zoonotic pathogens other than SARS-CoV-2 are still in their early stages. They can be challenging to implement due to their technical complexity, costs, and scalability. To address this issue, we examined the use of xeno-diagnostic tools (qRT-PCR) and RNA sequencing to characterize arboviruses present in wild mosquito populations in Brisbane, the state capital of Queensland, Australia. Our results reveal co-circulation of multiple arboviruses maintained by a wide range of vector species. Some of these are of considerable importance in a public health context. We also discuss how the monitoring approaches that we used might augment existing disease surveillance programs.

## 2. Methods

### 2.1. Study area

This study was carried out in Brisbane, Queensland (27°28ʹ12ʹ’ S and 153°01ʹ15ʹ’ E) between March 2021 and May 2022. Brisbane is the third most populous city and the largest state capital in Australia by geographic area. It has a subtropical climate, a rainy season from November to March (annual precipitation levels of 1011.5 mm) and monthly average temperatures of 10–22°C and 20–29°C in winter and summer, respectively (BOM 2023). The greater Brisbane area contains many habitat types including freshwater, estuarine wetlands, saltmarshes, mangroves, bushlands, and subtropical rainforests (Queensland Government 2023). Brisbane is Australia’s most biodiverse capital city with a variety of native and introduced wildlife, and arbovirus vector and reservoir species. In combination with the high human notification rates for BFV and RRV (Knope et al. 2019), this makes the city an ideal location to investigate endemic and emerging arboviruses that circulate in sylvatic/urban interfaces.

### 2.2. Study design

In Queensland, mosquito-based surveillance systems are used to survey a subset of arboviruses. High mosquito trap rates (>100,000 mosquitoes per week) and the logistical challenges of collecting mosquitoes at intervals short enough to preserve viral RNA made processing time-consuming, labour-intensive, and inefficient (van den Hurk et al. 2012). To address these challenges, sugar-based surveillance systems were implemented to detect pathogens in the saliva of infected mosquitoes (Hall-Mendelin et al. 2010, van den Hurk et al. 2014). These systems use sugar-baited filter papers [Flinders Technology Associates (FTA) cards®] to preserver nucleic acids and encourage mosquito feeding and expectoration. This system is currently utilized as part of a limited mosquito and arbovirus surveillance program funded by Queensland Health (QH) with major contributions from some local councils.

Our study was conducted in partnership with Brisbane City Council (BCC) and QH. BCC routinely deploys mosquito traps (light traps) containing honey-soaked FTA cards on a weekly basis, from October to May each year. QH screens the elutes from these retrieved FTA cards for notifiable arboviruses RRV, BFV, and JEV using specific TaqMan real-time RT-PCR assays, as described elsewhere (Hall-Mendelin et al. 2010, Hall et al. 2011, QH 2022). In this study, we were notified of positive FTA cards and retrieved the corresponding mosquito collections from BCC. In collaboration with QH, we then conducted additional trapping at the same sites to increase the number of (potentially) virus-infected mosquito pools for testing.

This targeted approach aimed to maximize the detection of virus-positive mosquitoes and obtain high-quality genomic data essential for understanding arbovirus genotypes and their relationships with mosquito vectors in Southeast Queensland. FTA cards served as a preliminary screening tool, indicating likely infection and high viral concentrations. Testing expectorated saliva from FTA cards allowed us to assess transmission potential, enhancing the effectiveness of the RNA sequencing approach in characterizing arbovirus genomes and inferring mosquito vectors.

### 2.3. Mosquito collection and identification

**Figure 1.**
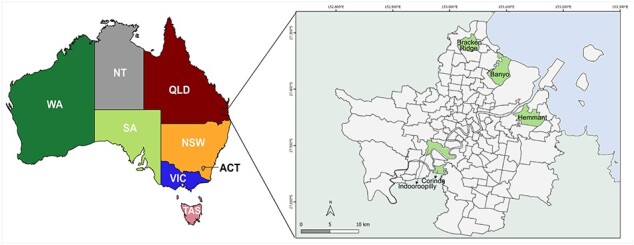
Map of Australia and study area in southeast Queensland.

Five BCC trap sites representing a diversity of mosquito habitats were chosen. All were urban parks near residential areas. Three sites were within 500 m of a saltmarsh, and the other two sites were close to freshwater habitats (Table 1, Fig. 1). CDC-style light traps (Pacific Biologics, Scarborough, Australia) were baited with 2 kg of dry ice as a CO_2_ source. Traps deployed by BCC were also baited with 1-octen-3-ol (Merck Life Science, Bayswater, Australia) (van Essen et al. 1994). Traps were set once a week prior to dusk and then collected the following morning. The mosquitoes from each trap were transported to the Mosquito Control Laboratory, where they were cold-anesthetized, identified using dichotomous keys (Marks 1982, Russell 1993, 1996) and pooled (up to 200 individuals) according to species, site, and collection date. Mosquito pools were stored at −80°C until RNA extraction. We verified and confirmed the morphological identification, as detailed in Johnson et al. (2024), by targeting the mitochondrial cytochrome oxidase subunit 1 (COI) (Leray et al. 2013) and the second internal transcribed spacer (ITS2) of nuclear ribosomal DNA (Batovska et al. 2017).

**Figure 2.**
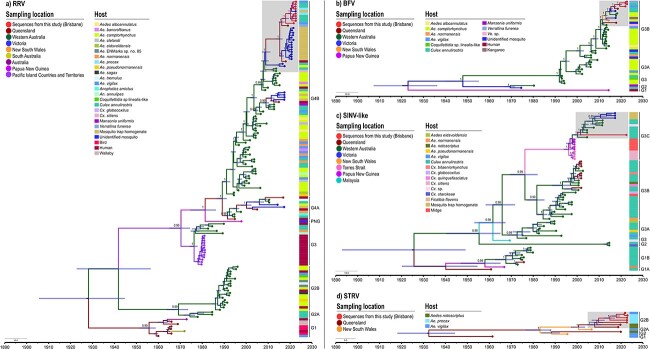
Maximum clade credibility tree based on the phylogenetic analysis of complete coding region of (a) RRV, (b) BFV, (c) INV-like, and (d) STRV.

The eight Australian states and territories (ACT—Australian Capital Territory; NSW—New South Wales; NT—Northern Territory; QLD—Queensland; SA—South Australia; TAS—Tasmania; VIC—Victoria; WA—Western Australia) are shown on the left, color-coded according to the phylogenetic analysis in this study (see Fig. 2), with the inset marking in green the five suburbs containing the collection sites.

**Table 1.** Mosquito trap site location, type, and social characteristics of Brisbane local government area, Queensland, Australia

Site name, suburb	Geocordinates	Ecosystem(s) classification	Dominant habitat type	Human pop. density^a^ (2021)
Bracken Ridge	27.307225° S, 153.040433° E	Metropolitan, wetland	Saltmarsh	21.56 people/ha
Indooroopilly	27.511639° S, 152.984458° E	Metropolitan, wetland	Suburban, riparian	18.26 people/ha
Corinda	27.549861° S, 152.994836° E	Metropolitan, wetland	Suburban, freshwater	18.45 people/ha
Banyo	27.369166° S, 153.072694° E	Metropolitan, coastal	Saltmarsh	7.75 people/ha
Hemmant	27.451706° S, 153.123781° E	Metropolitan, coastal	Saltmarsh	12.77 people/ha

Human population size and density are derived from the ^a^Australian Bureau of Statistics 2022 census (Australian Bureau of Statistics 2022).

### 2.4. Sample preparation and nucleic acid extraction

Mosquito pools were homogenized in 2.0 ml Eppendorf Safelock microcentrifuge tubes using DNA/RNA Shield storage buffer (Zymo Research, Irvine, USA) and 2.3 mm zirconium silica beads (Daintree Scientific, St Helens, Australia) scaled according to the mosquito numbers present in each sample (volumes presented in Supplementary Table S1). Mosquito samples were mechanically homogenized for two 3-min cycles at 1500 rpm using a Mini Beadbeater-96 (BioSpec Products, Bartlesville, USA) and centrifuged for two 5-min cycles at 14,000 rpm, at 4°C. Nucleic acid was extracted from the supernatant using a QIAamp Viral RNA Mini Kit (Qiagen, Hilden, Germany) following the manufacturer’s instructions with modifications. The carrier RNA was not added, and a double elution of 40 µl each was performed using UltraPure water (Invitrogen, Carlsbad, USA). An extraction negative control using DNA/RNA Shield was included in each batch of extractions. Extracted nucleic RNA was stored at −80°C until further processing.

### 2.5. Viral detection

Pools were tested for the presence of flaviviruses and alphaviruses using qRT-PCR with genus-specific primers targeting the NS5 and nsP4 regions, following Vina-Rodriguez et al. (2017) and Hermanns et al. (2017) protocols, respectively, with modifications outlined below. Briefly, a SYBR green I based qRT-PCR kit (Luna Universal One-Step NEB, Ipswich, USA) was used in PCR mixtures (20 µl) containing 2 µL cDNA, 10 µL 2X Luna Universal One Step master mix, 0.8 µl WarmStart Luna RT, 5.6 µL UltraPure water, and 0.4 µM (final concentration) of forward and reverse primers designed to either amplify flaviviruses—Pflav-fAAR (TACAACATGATGGGAAAGAGAGAGAA RAA) and PflavrKR (GTGTCCCAKCCRGCTGTGTCATC) 243 bp region—or to amplify alphaviruses—Pan-Alpha-F1 (TCAGCAGAAGAYTTYGAYGC) and Pan-Alpha-R2 (ACATTCCAGAYTTCATCAT) 253 bp region. Thermocycling consisted of 1 cycle of reverse transcription at 55°C for 10 min, followed by RT inactivation/polymerase activation at 95°C for 1 min, then 40 cycles of amplification at 95°C for 10 s, 50°C for 20 s, and 60°C for 30 s (data collection). PCR amplification was carried out using the MIC platform (Biomolecular Systems, Sydney, Australia).

For both primer sets, a negative extraction control (see Section 2.4), a no template control, and serial 10-fold dilutions of known concentrations of positive controls [CHIKV for alphavirus tests and DENV for flavivirus tests—kindly provided by Dr Narayan Gyawali, QIMR Berghofer (QIMRB)] were included for melting curve analysis. Amplicons with well-defined melting curve peaks matching the expected range for different alpha- or flaviviruses (*n* = 30 amplicons from 28 samples) were sent to the QIMRB’s Analytical Facility and confirmed by Sanger sequencing (ABI-PRISM 3130 Genetic Analyser, Applied Biosystems, Foster City, USA). Sequences were compared with published sequences using Basic Local Alignment Search Tool and the GenBank database to confirm the identity of the virus. A subset of samples returned detections of medically important arboviruses (*n* = 20 samples) and was then submitted for library preparation, using an RNA sequencing approach to generate whole genomes of the already identified viruses. The remaining samples (*n* = 8) returned hits against either insect-specific viruses or unclassified viruses and were not further investigated in this study.

### 2.6. Library preparation and RNA sequencing

Extracted samples were DNase treated (NEB, Ipswich, USA) to eliminate any residual DNA molecules from the host in the RNA extracts. After DNase treatment, samples were assessed for RNA quality using the Agilent 2200 TapeStation system (Agilent Technologies, Santa Clara, USA) and subsequently quantified with the Qubit 3.0 fluorometer (Qubit RNA HS and dsDNA HS assay kits; Thermo Fisher Scientific, Waltham, USA) to standardize RNA for Illumina library preparation. All samples had an RNA integrity number of >3.0. In the library preparation, a library negative control was used, comprising 20 µl of UltraPure water (Invitrogen, Waltham, USA). For a positive control library, 20 µl of RNA from a pool of 1000 mosquitoes containing a single RRV-infected mosquito was included, as recommended (Batovska et al. 2019). A total of 20 µl of undiluted DNase treated RNA extracts with the amount of RNA ranging from 50 to 500 ng were used to construct sequencing libraries with the Ovation universal transcriptome sequencing (RNA-Seq) system (NuGEN, San Carlos, CA) with customized mosquito rRNA depletion probes (Batovska et al. 2019). Paired-end (150-bp) sequencing of each library (approx. 20 million reads per library) was then performed on the NextSeq 550 platform (Illumina). All library preparation and sequencing procedures were carried out by the Analytical Facility at QIMRB.

### 2.7. Genome assembly and annotation

Reads were assembled using the ViralFlow pipeline (Dezordi et al. 2022), a workflow that performs reference-based genome assembly along with several complementary analyses. Source code and detailed documentation for ViralFlow can be found at https://viralflow.github.io/index-en.html. Briefly, FastQC was used to assess the quality of Illumina raw reads, and low-quality reads were trimmed, and high-quality reads were mapped against a reference genome using BWA v.0.7.17 (Li and Durbin 2009) with default settings, limiting single nucleotide polymorphisms (SNPs) to 3 per 150 bp fragment. Following alignment, BAM files were sorted and indexed using Samtools (Li et al. 2009). Minor variant analyses were performed using Samtools v.1.9 and iVar v.1.3.1 (Grubaugh et al. 2019a) for the accurate recovery of SNPs and indels. Two consensus sequences were generated: one based on the predominant allele at each nucleotide position across the genome and another incorporating ambiguous nucleotide characters where intrahost single nucleotide variants made up to 60% of allele frequencies. Only high-quality mapped bases (score ≥30) were considered, and positions with a minimum depth of coverage threshold of 100× were required to identify supported intrahost variants. Consensus sequences of viral genomes were obtained through the Integrated Genome Viewer software (Robinson et al. 2011). Additionally, reads were de novo assembled into contigs using metaSpades (Nurk et al. 2017), as previously described previously by da Silva et al. (2024). The resulting sequences were compared to the consensus generated using ViralFlow, and no differences were observed between them.

### 2.8. Bioinformatic analyses

Coding regions of the complete genomes generated in this study were aligned with all published near-complete genomes (>10 kb). Sequences obtained from public databases that presented duplicate strain names for the same strain or that corresponded to cell passages of the same original isolate were removed. All genomes and data (date of isolation, host, and location) were collected from ViPR (Pickett et al. 2012): http://www.viprbrc.org/, accessed on 10 September 2023 (accession numbers and genomic information given in Supplementary Table S2). Sequences that had no data described were manually assigned through literature searches. Multiple sequence alignments were performed using MAFFT v7 (Katoh et al. 2019): http://mafft.cbrc.jp/alignment/software/, and manually edited in AliView v1.26 (Larsson 2014).

Potential recombination events were investigated using the full set of tests implemented in Recombination Detection Program—RDP v4.101 (Martin et al. 2015) and genomes showing recombination events supported by at least three of the seven methods were removed from further analyses (Langat et al. 2020).

### 2.9. Phylogenetic analyses

The optimal evolutionary model was selected using the Akaike information criterion in Smart Model Selection (SMS) (Lefort et al. 2017) implemented in PhyML v3.0 (Guindon et al. 2010) http://www.atgc-montpellier.fr/phyml/. Tamura-Nei (TN93) with gamma distributions (+G) (TN93 + G) gave the best fit for Stratford virus (STRV), general time-reversible (GTR) model with gamma distributions (GTR + G) for BFV and Sindbis-like virus (SINV), and with invariant sites (+I) (GTR + G + I) for RRV. Maximum-likelihood (ML) phylogenies were generated using PhyML v3.0, employing a Subtree Pruning and Regrafting topology searching algorithm. We assessed statistical support for phylogenetic branching points using the approximate likelihood ratio test on the Shimodaira-Hasegawa-like procedure (SH-aLRT) with 1000 replicates.

### 2.10. Molecular clock

To evaluate if the data were appropriate for the estimation of temporal parameters, a regression analysis of the root-to-tip divergence against tip sampling time of the ML phylogenetic trees was performed in TempEst v1.5.3 (Rambaut et al. 2016) (Supplementary Table S3). Outlier sequences that deviated by > 1.5 interquartile ranges from the root-to-tip regression line were excluded. We found strong temporal signals (correlation coefficients ranging from 0.8623 to 0.9758 and *R*^2^ ranging from 0.6404 to 0.9521), suggesting that the different datasets were appropriate for the estimation of temporal parameters. Bayesian molecular clock phylogenetic analysis was performed for each virus with BEAST 1.10.4 (Suchard et al. 2018) in three independent runs of 100 million Markov chain Monte Carlo, sampling every 10,000 generations. After removing 10% burn-in, the final Bayesian consensus tree datasets were generated. The molecular clock and demographic model were chosen by identifying the optimal likelihood combination through Path Sampling/Stepping-stone sampling. This comparison involved evaluating strict and uncorrelated lognormal clock models, each associated with two distinct demographic models: Constant and Bayesian Skyline priors (Supplementary Table S4). Convergence between runs was evaluated with Tracer 1.7.1 (Rambaut et al. 2018) and the final combined dataset showed an effective sample size > 200 for all parameters sampled. The tree files of each virus were combined using LogCombiner v1.10.4 and maximum clade credibility (MCC) trees were extracted and summarized using TreeAnnotator v1.10.4. Tree visualization and figure generation were performed with FigTree v1.4.4 (Rambaut 2014). For phylogeographic analysis, we employed Bayesian phylogenetic methods, specifically using MCC annotated trees derived from the complete coding region of the arboviruses to summarize inferred geographic diffusion patterns. SPREAD4 software (Nahata et al. 2022) (https://spreadviz.org/) was used to create geographic maps that allowed the visualization of discrete and continuous spatio-temporal reconstructions and geographic migration history, thereby enhancing our understanding of the geographic spread of the viruses.

## 3. Results

### 3.1. Mosquito diversity

We were notified of 11 traps collected between March 2021 and May 2022 in our 5 sampling areas that had FTA cards positive for RRV. We recovered the contents of those traps and deployed a total of 11 additional samplings at the same sites, totalling 22 traps. A total of 54,670 adult mosquitoes from 26 species were collected. Species abundance and diversity differed between sites, with greatest overall diversity of species in Banyo (23 species), a site with a mix of freshwater, saltmarsh, and estuary, compared with the least diverse site, located in Hemmant (7 species), an industrial suburb. Mosquito abundance was also greatest in Banyo (47.4% of all mosquitoes). The dominant species across all sites were *Culex annulirostris* (53.5%), *C. orbostiensis* (14.9%), and *A. procax* (8.1%) (Supplementary Table S5).

### 3.2. Viral screening and RNA sequencing

Mosquitoes were separated into 382 species-specific pools, each pool containing up to 200 mosquitoes. Of these, a total of 30 virus detections were made from 28 pools using qRT-PCR. All detections were confirmed by Sanger sequencing. We detected four medically relevant arboviruses: the alphaviruses BFV, RRV, and Sindbis-like virus (SINV-like), and the flavivirus Stratford virus (STRV). Detections were made from seven mosquito species, mostly *C. annulirostris* and *A. procax* (Table 2).

**Table 2.** Details of viruses detected in mosquitoes collected from Brisbane sites, from March 2021 to May 2022

Pool no.	Species	Collection site	Date	QTD	qRT-PCR result/confirmed by Sanger sequencing	RNAseq result	No. total reads	No. mapped reads	% mapped reads	Depth of coverage	Sequence length (nt)	Reference genome	Genome reference length (nt)
BCC 16	*Aedes vigilax*	Bracken R	15/3/2021	161	RRV	RRV	40,926,873	1,003,045	2.45	6311	11,913	MH987781.1	11,920
BCC 32	*Culex annulirostris*	Bracken R	5/1/2022	200	RRV	RRV	40,836,524	1,474,766	3.61	9279	11,911	MH987781.1	11,920
BCC 45	*Aedes procax*	Corinda	17/1/2022	160	RRV	RRV	40,000,705	881,589	2.20	5547	11,911	MH987781.1	11,920
BCC 48	*Aedes procax*	Corinda	7/2/2022	117	RRV	RRV	40,922,299	390,761	0.95	2459	11,912	MH987781.1	11,920
BCC 63	*Culex annulirostris*	Indooroopilly	14/2/2022	135	RRV	RRV	35,404,670	275,631	0.78	1734	11,912	MH987781.1	11,920
BCC 70	*Culex annulirostris*	Banyo	21/2/2022	200	RRV	RRV	39,348,668	963,678	2.45	6063	11,911	MH987781.1	11,920
BCC 83	*Aedes vigilax*	Banyo	11/4/2022	104	RRV	RRV	42,975,529	625,696	1.46	3937	11,911	MH987781.1	11,920
QH 138	*Culex annulirostris*	Bracken R	4/2/2022	200	RRV	RRV	46,412,925	622,180	1.34	3915	11,912	MH987781.1	11,920
QH 329	*Culex annulirostris*	Indooroopilly	9/3/2022	200	RRV	RRV	41,290,576	60,550	0.15	381	11,908	MH987781.1	11,920
BCC 380	*Culex orbostiensis*	Hemmant	17/5/2021	1	RRV	No sequence obtained	40,110,924	-	-	-	-	-	-
BCC 317	*Culex sitiens*	Indooroopilly	14/2/2022	3	STRV & RRV	No sequence obtained	35,639,262	-	-	-	-	-	-
QH 160	*Verrallina funerea*	Bracken R	4/2/2022	58	BFV	BFV	40,821,131	2,974	0.01	19	11,549	MN064696.1	11,574
BCC 71	*Culex annulirostris*	Banyo	21/2/2022	200	BFV	BFV	39,117,880	4,476	0.01	29	11,554	MN064696.1	11,574
BCC 22	*Aedes vigilax*	Bracken R	24/5/2021	125	BFV	BFV	41,718,056	49,426	0.12	320	11,555	MN064696.1	11,574
						STRV	41,718,056	3,657	0.01	25	10,830	MZ358850.1	10,846
BCC 76	*Aedes procax*	Banyo	11/4/2022	200	STRV	STRV	40,014,774	38,728	0.10	268	10,846	MZ358850.1	10,846
QH 158	*Aedes procax*	Bracken R	4/2/2022	202	STRV	STRV	43,652,133	2,153	0.005	15	10,803	MZ358850.1	10,846
QH 226	*Aedes procax*	Banyo	3/2/2022	132	STRV	STRV	39,253,971	2,326	0.10	16	10,846	MZ358850.1	10,846
QH 295	*Aedes procax*	Corinda	17/2/2022	190	STRV	STRV	32,878,765	15,082	0.05	104	10,846	MZ358850.1	10,846
BCC 67	*Culex annulirostris*	Banyo	21/2/2022	200	SINV	SINV	34,843,064	6,429	0.02	42	11,604	OP950209.1	11,611

Depth of coverage = Number of mapped reads × 75 (read length)/reference genome length. QTD: quantity.

BCC (collected by Brisbane City Council) = samples with previous detection of RRV in FTA cards.

QH (collected by Queensland Health) = samples collected after detection of RRV in FTA cards.

For the 20 mosquito pools further investigated by RNA sequencing analysis, a mean of 39,622,773 paired reads was generated per sample (range: 32,878,765–46,412,925). We generated 18 novel full-length sequences, with some samples containing up to two different viruses (Table 2). Full-length genomes displayed an average coverage depth of 2248 reads (range: 15–9279), with genome size ranging from 10.803 to 11,913 nucleotides (Table 2).

### 3.3. Phylogenetic analyses

The 18 complete genomes generated in this study (9 of RRV; 3 of BFV; 1 of SINV-like; and 5 of STRV) were used to reconstruct the phylogenetic relationship of these viruses in comparison with the genomic sequences available in public databases. Overall, phylogenetic analysis suggested continuous circulation of all arboviruses over the last 90 years in Australia. The lineages that are currently circulating in the country are RRV_G4B, SINV_G3C, STRV_G2B, and BFV_G3B, which all emerged within the last 26 to 11 years (Fig. 2 and Supplementary Fig. S2). Phylogeographic analysis revealed long-distance dispersal of all lineages within Australia (Supplementary Table S6; Supplementary Fig. S1).

Nodes are colored based on their geographical origin, as indicated in the map of Australia showed previously. Colored squares represent host origin. Gray shaded squares represent clades circulating in the study area. Posterior probability values of >0.80 are presented above main branches.

Except for STRV, all the other viruses investigated showed a clear ladder-like tree structure (Fig. 2), which demonstrates that different epidemiological, immunological, and evolutionary processes may actively shape the evolution of Australian arboviruses.

#### 4.3.1. Ross river virus

A total of 141 RRV whole-genome sequences from publicly available databases were analyzed, including the nine new genomes generated here. The virus sequences originated from PNG, the PICTs and different states of Australia, including New South Wales (NSW), Queensland (QLD), South Australia (SA), Victoria (VIC) and Western Australia (WA) (Supplementary Fig. S3). A ML phylogeny was re-constructed showing that the sequences obtained in this study belonged to the G4 genotype, which encompasses all contemporary (1994–2022) RRV isolates (Michie et al. 2021). Our samples belonged to the G4B lineage, which includes mosquito-derived strains from QLD, VIC, and WA, and human-derived strains from QLD and WA (Supplementary Fig. S4).

The time to the most recent common ancestor (TMRCA) of all RRV genomes was estimated to be around 1927 (95% HPD = 1908 to 1943) and the current subclade of G4B has been circulating since ∼ 2007 (95% HPD = 2005 to 2008) (Fig. 2a).

#### 4.3.2. Barmah forest virus

A total of 39 BFV whole-genome sequences were analyzed. The strains were sampled from PNG and from the Australian states of NSW, QLD, VIC, and WA, between 1974 and 2018, including our three new genomes from 2021 to 2022 (Supplementary Fig. S5). The sequences obtained in this study belong to G3 genotype, G3B lineage, to which all contemporary BFV strains in Australia belong (Michie et al. 2020). This lineage consists of mosquito-derived strains from QLD, VIC, and WA, and a kangaroo-derived strain from NSW (Supplementary Fig. S6). The TMRCA of all BFV genomes was estimated to occur around 1922 (95% HPD = 1907 to 1936) and the current subclade of G3B emerged around 2011 (95% HPD = 2010 to 2012) (Fig. 2b).

#### 4.3.3. Sindbis-like virus

All available whole and draft genome (>10 kb) sequences of Sindbis and Sindbis-like viruses were analyzed. The final alignment contained 161 sequences from Australasia, Africa, and Europe, described between 1953 and 2022 (Supplementary Fig. S7). Phylogenetic analyses revealed four major genetic groups with more than 23% nucleotide sequence divergence and 8% amino acid sequence divergence between these groups, across their entire genomes. According to the International Committee on Taxonomy of Viruses, this meets the criteria for demarcating Alphavirus species (Powers et al. 2009). These new species have been described by Michie et al. (2023).

Our sample belongs to the most recent lineage of SINV-like present in Australia, known as G3C (Supplementry Fig. S8). This lineage consists of mosquito-derived strains, mostly from *C*. *annulirostris*, from QLD, VIC, and WA, and midge-derived strains from PNG (Supplementary Fig. S9). The TMRCA of all SINV-like lineages of Australia was estimated to be approximately 1924 (95% highest posterior density (95% HPD = 1886 to 1949). The emergence of G3C dates to approximately 1996 (95% HPD = 1994 to 1998), and the current subclade has been circulating since 2000 (95% HPD = 1998 to 2002) (Fig. 2c).

#### 4.3.4. Stratford virus

A total of 11 STRV whole-genome sequences sampled from NSW and QLD, between 1961 and 2019, including the five new genomes generated in this study (from 2021 to 2022) were analyzed (Supplementary Fig. S10). Despite the analysis being limited to only eleven STRV isolates, phylogenetic analyses revealed genetic diversity in mosquito-derived samples from QLD and NSW. The TMRCA of STRV was estimated around 1932 (95% HPD = 1918 to 1944). The current subclade, G2B, has been circulating since 2007 (95% HPD = 1997 to 2003) (Fig. 2d).

## 4. Discussion

Arboviruses pose a significant threat globally. There are more than 500 known arboviruses of which approximately 100 are pathogenic to humans (Artsob et al. 2024). In Australia, at least 13 arboviruses have been associated with human disease. Some of the endemic zoonotic arboviruses, such as BFV and RRV, are maintained in complex, poorly understood transmission cycles, involving a broad range of mosquito species and potential vertebrate hosts (Mackenzie et al. 1994, Ong et al. 2021). As a result, transmission pathways may differ geographically and temporally, leading to variable human transmission risks. Here, we successfully generated 18 novel full-length genome sequences, the phylogenetic analysis of which increased our current understanding of Australian arbovirus evolution. These sequences and their analyses highlight major knowledge gaps in arbovirus phylogeny in Australia and unveil an evolutionary history marked by continuous transmission, co-circulation, and steady lineage modification over time.

The arboviruses found in our study are widespread within Australia. While their exact geographic origins and route of spread could not be determined due to limited whole-genome sequence information, phylogeographic analysis indicated instances of long-distance movement of all lineages within the country probably facilitated by the movement of viraemic hosts (including mosquitoes, birds, humans, and other mammals). Isolates from widely separated locations shared similar genomic sequences and lineages, and suggested evolution over a large temporal timeframe (100 years) (Fig. 2 and Supplementary S1). Genomes sequenced by this project were recovered from *C. annulirostris, A. vigilax, A. procax*, and *V. funerea*. All are competent vectors of at least a subset of the viruses isolated (Harley et al. 2001) and might play key roles in establishing local transmission across parts of their ranges. However, further studies, and many more genomes, are needed to better understand the patterns of circulation and turnover of these virus lineages and to characterize the role of individual reservoirs and vector species in maintaining the transmission of specific arbovirus lineages in different habitats.

The phylogenies of Australian alphaviruses exhibit a ladder-like structure, indicating the persistence of a dominant lineage and suggesting immune-driven evolution, similar to patterns observed in DENV and ZIKV, which have undergone significant evolution and diversification driven by positive selection in their entire polyproteins, also subject to intense directional evolution (Durães-Carvalho et al. 2019). DENV also faces immune pressures from both human and mosquito hosts, resulting in novel variants (Wash and Soria 2015, Stica et al. 2022). However, the inherent complexity of arbovirus phylogenetic trees, due to many components of transmission, complicates the analysis of evolutionary rates and selective pressures, particularly in zoonotic viruses like RRV, which involve multiple vectors and reservoirs. Consequently, purifying selection may also play a significant role by preserving viral fitness across diverse hosts, preventing over-specialization to any single host (Lequime et al. 2016). For instance, DENV intra-host genetic diversity within mosquito vectors is primarily shaped by genetic drift and purifying selection (Lequime et al. 2016), a pattern also observed in WNV, where avian infections help maintain genetic variation despite frequent host switching (Jerzak et al. 2008). This duality highlights the complex evolutionary pressures acting on arboviruses and that both purifying and positive selection pressures may actively shape their evolution. Therefore, the long-term transmission of arboviruses in Australia, combined with high seroprevalence in multiple vertebrate host species (Vieira et al. 2024), further supports the influence of both selection pressures, at least for BFV, RRV, and SINV.

While phylogenetic trees serve as useful indicators of epidemiological, immunological, and evolutionary processes affecting viral genetic variation, ladder-like trees (e.g. Fig. 2a–c) can indicate the presence of directional selection, but they can also reflect the sequential genetic bottlenecks (genetic drift) associated with rapid spatial spread (e.g. rabies virus; Streicker et al. 2010). In such cases, genetic drift may enable random viral variants to persist during transmission bottlenecks, independent of selective pressures. To further understand phylogenetic tree shapes and test these hypotheses, more genomic surveillance data is required. Exploring these factors would provide valuable insights into arboviral transmission dynamics.

Similar divergence dates were estimated for all the arboviruses identified in this study, with genotypes diverging around 1922–1932. Coincidentally, this timeframe aligns with the first reports of “an unusual epidemic” in Australia, with the syndrome of polyarthralgia and rash (Nimmo 1928). The causal agent of the disease was later suggested to be RRV (Doherty et al. 1964, 1971), but could have been caused by any “indigenous pathogen” (Jacups et al. 2008). While some major ecological event may have led to the emergence of arboviruses at this time, we also note that 100 years may simply represent the limits of our methodology: rapid viral evolution allows us to recover past estimates of viral emergence and divergence but once signal saturation is reached it limits accurate estimates of ancient viral emergence at the distant past (Aiewsakun and Katzourakis 2015).

### 4.1. Virus screening and phylogenetic analyses

RRV is responsible for the highest number of human arbovirus infection notifications across every state and territory of Australia (Jansen et al. 2019) (Supplementary Fig. S11). The virus was first isolated in 1959, from a pool of *A. vigilax* mosquitoes collected from Queensland (Doherty et al. 1963b) and from humans in 1972 in the same state (Doherty et al. 1972). Over 40 species of mosquito and 20 vertebrate hosts have been associated with RRV transmission (Claflin and Webb 2015, Stephenson et al. 2019). Sixty-five years after its isolation, Australia remains no closer to understanding transmission, predicting human spillover, or implementing a surveillance program that could enhance our understanding. In this study, five mosquito species were found to harbor RRV, four of which have been previously identified as competent RRV vectors in laboratory studies (reviewed in Russell 2002). Of these, *A. vigilax* and *C. annulirostris* yield the most field detections and vector competence studies have implicated these as vector species, along with *A. procax* and *C. sitiens*. We also detected RRV by PCR in *C. orbostiensis*, but no laboratory studies on the vector competence of this species have been conducted.

Phylogenetic reconstructions were made using RRV sequences from 20 mosquito species, birds, humans, and wallabies. All belonged to the G4 genotype, G4B lineage. As described by Michie et al. (2021), all strains collected in Australia since 1996 belong to G4 genotype, indicating that it is the contemporary and dominant genotype in circulation in the country. The last Australian detection of G4A occurred in 2016 (VIC and QLD) and all recently sampled strains, including sequences from QLD (2016–18), VIC (2016–17), WA (2008–13), and this study (2021–22), were classified as G4B. The G4B lineage have been found in samples from humans and 15 mosquito species, which underscores the complexity of RRV’s vector range but there are no sequences from nonhuman vertebrate hosts, highlighting a key gap in knowledge regarding reservoir incrimination.

BFV is responsible for the second highest number of human arbovirus notifications in Australia (Knope et al. 2019) (Supplementary Fig. S12). This alphavirus was first isolated in 1974 from *C. annulirostris* mosquitoes collected from VIC (Marshall et al. 1982) and concurrently from mosquitoes trapped in Queensland (Doherty et al. 1979). The first case of BFV infection in humans was not reported until 1986 (Boughton et al. 1988). The virus has been isolated from several wild-caught mosquito species, including those found positive for the virus in this study: *C. annulirostris*, *A. Vigilax*, and *V. funerea* (Jeffery et al. 2006, Jacups et al. 2008). The sequences obtained in this study belong to the G3 genotype, G3B lineage. G3 is the contemporary genotype circulating in Australia (Michie et al. (2020)) and the G3B is widely distributed. The number of sequences of the G3B lineage is limited but originate from a kangaroo and six mosquito species. More genomic information is needed to understand the potential transmission dynamics of this virus.

Sindbis virus (here called Sindbis-like virus due to its divergence from the Europe-African virus group; Michie et al. 2023) is an alphavirus first isolated in Australia in 1960 from *C. annulirostris* mosquitoes (Doherty et al. 1963a). Approximately 65% of SINV-like genomes investigated in Australia were isolated from this mosquito, including the strains found in this study. It is one of the most commonly isolated arboviruses in Australian mosquitoes (Ong et al. 2021). The genotype detected in this study, G3C, circulates in WA and has also been detected in mosquitoes from VIC in 2016 (Batovska et al. 2022). Although SINV-like virus appears to have an association with mild infection in humans (Doherty et al. 1969, Doherty 1973, Guard et al. 1982), the resulting health implications remain unclear, and its vertebrate reservoirs are unknown. No outbreaks of SINV-like virus have been reported in Australia, despite clear evidence that this virus has been circulating in the country for over a century. It is not notifiable in the country and there is no clinical or widely available laboratory diagnostic.

Stratford virus was the only flavivirus found in our study. It is a member of the Kokobera virus subgroup and was first isolated in 1961 from *A. vigilax* mosquitoes collected in north Queensland (Doherty et al. 1963a). It is often detected in *Aedes* species, but information regarding vertebrate reservoirs is not available (Toi et al. 2017). Based on serological evidence, sporadic human infections with STRV have been documented in asymptomatic individuals in NSW (Hawkes et al. 1985), as well as in symptomatic patients in NSW and QLD with symptoms including fever, joint pain, and lethargy (Phillips et al. 1993, Johansen et al. 2005, Pyke et al. 2021). Notifications designated as “unspecified-flavivirus” infections, which includes STRV, are frequently submitted to the Australian Government National Notifiable Disease Surveillance System. These cases are confirmed through serology, and about 30 unspecified-flavivirus infections are reported annually reaching its peak (116 cases) in 2016 (Australian Government 2024) (Supplementary Fig. S13). Between 2017 and 2020, a total of 49 suspected-flavivirus infections in QLD were STRV IgM positive (Pyke et al. 2021).

Although limited to a comparison of 11 available contemporary STRV full sequences, phylogenetic analysis demonstrated genetic diversity among the respective QLD and NSW isolate groups. Together with our six new isolations in Brisbane, these findings support evidence of ongoing circulation of STRV. The geographical distribution of the virus is likely to be broader than currently described and potential vertebrate reservoirs remain unknown. Further genomic surveillance would improve our understanding of STRV circulation.

### 4.2. The role of xeno-monitoring coupled with RNA sequencing in disease surveillance

Xeno-surveillance of mosquito-borne pathogens in Australia has been underway for many decades, but state programs differ in magnitude and by methodological sensitivity. Routine screening of mosquitoes (by trap homogenate or by FTA card) only occurs for a subset of “notifiable” Australian endemic viruses: BFV, RRV, JEV, WNV_KUN_, and MVEV (Australian Government 2024). These are PCR tests for virus fragments, often in undifferentiated mosquito pools (i.e. no data on species associations) and, routinely, detections are not investigated by genome sequencing. For instance, during the 2022–23 surveillance season, a total of 179 arbovirus detections in mosquitos were recorded in the states of NSW, VIC, and SA (Government of South Australia 2023, NSW Government 2023, Victoria State Government Department of Health 2023) (Supplementary Table S7), but no genomic data are available. No public reports on xeno-surveillance programs from other states are available. Non-notifiable arboviruses circulating in Australia, including SINV-like and STRV, are not routinely tested for using xeno-diagnostic surveillance, possibly because, to date, their pathogenicity in humans appears mild and is poorly described. In the absence of comprehensive surveillance programs, current xeno-surveillance programs tend to simply identify the presence of viruses in complex environments that are already known to be endemic. The key transmission pathways and distributions of specific genetic lineages remains unknown (Gyawali et al. 2019). Nonetheless, there is increasing awareness of the crucial nature of molecular xeno-monitoring in response to the emergence and re-emergence of neglected arboviruses (Hill et al. 2023, Laiton-Donato et al. 2023). This is of particular significance on the Australian continent where a large number of ecosystems, at huge geographic scales, are under constant environmental disturbance potentially favoring arbovirus emergence and dispersal through dynamic changes in mosquito vector populations, reservoir distribution, and human proximity to those reservoirs and vectors. Designing a sustainable but informative surveillance system is a tremendous challenge.

The COVID-19 pandemic has highlighted the utility of genomic surveillance in tracking and monitoring the spread of viruses, detecting new variants, and informing public health interventions (Hill et al. 2023, Zeghbib et al. 2023). However, adapting routine sequencing into public health investigations will require additional programmatic investment in expertise and resources. We demonstrate that pan-alphavirus and pan-flavivirus screens can be used to test large but differentiated mosquito pools for arboviruses. This methodology not only enhances current arbovirus surveillance but also holds potential for broader application within a One Health framework, integrating data from human, animal, and environmental health to better understand zoonotic disease dynamics. Further investigations on genomes are then conducted on positive samples. Sequencing the “known” positives reduces the costs associated with NGS and enhances the efficiency of the surveillance network, yielding more informative results. In the future, multi-locus DNA metabarcoding approaches, similar to those demonstrated in conservation science (Arulandhu et al. 2017), might further increase efficiencies by initial screening that includes the identification of vectors, vertebrate signals, and pathogens from the same trap collection (Arulandhu et al. 2017, Johnson et al. 2024).

Whole-genome sequencing will improve existing information available for public health. It provides significant phylogenetic resolution that enables the reconstruction of local transmission chains, determination of the geographical origin of virus emergence and transmission, tracking of virus mutations, and identification of viral strains with modified phenotype (Pollett et al. 2020). Viruses such as RRV, BFV, JEV, and MVEV, are primarily maintained by active infection in animal reservoirs, which may, especially in the case of RRV, include humans (Supplementary Fig. S14 and S15) (Mackenzie et al. 1994). Although these viruses have encephalitogenic and arthritogenic symptoms of public health importance (see Mcguinness et al. 2023 for JEV and MVEV review, and Ong et al. 2021 for RRV review), there is limited whole-genome information for each virus, especially for MVEV and JEV in Australia (Supplementary Fig. S16 and S17, respectively). The introduction of genomic xeno-monitoring can significantly transform public health approaches by uncovering ecological factors that contribute to outbreaks, including cryptic transmission of viral lineages and transmission patterns (Cameron and Ramesh 2021). Ultimately, effective genomic xeno-monitoring programs have the potential to generate information for building risk models that can target public health messaging, vector control, and vaccination to prevent and mitigate the impact of arboviruses (Grubaugh et al. 2019b). Beneficiaries of this initiative would include public health agencies that can leverage better data for decision-making, communities that would benefit from enhanced vector control measures, and researchers who could gain insights into arboviral transmission dynamics (Hill et al. 2023, Laiton-Donato et al. 2023).

## 5. Conclusion

The simultaneous circulation of multiple arboviruses and a limited understanding of temporal transmission dynamics indicate that improved, well-designed surveillance programs of arboviruses, including genomic surveillance, are needed. Curation of longitudinal, consistent data sets that are shared with the scientific community is invaluable (Cameron and Ramesh 2021, Laiton-Donato et al. 2023). To gain a comprehensive understanding of the phylogeography and movement patterns of Australian arboviruses across the country, routine sampling and sequencing of viruses from different states and territories over time are necessary. This analysis would enable the identification of potential foci for viral diversity generation and the detailed source and sink hubs for transmission within Australia and the region. Finally, integrating sequencing approaches into arbovirus surveillance strategies around Australia may provide high-resolution data for researchers and public health agencies to better understand the emergence and evolution of viruses, ultimately enhancing preparedness and response strategies to mitigate the impact of future outbreaks.

## Supplementary Material

veae103_Supp

## Data Availability

Genome sequences obtained in this study have been deposited in GenBank under accession numbers PP496979 to PP496996, and the raw NGS reads are available in the NCBI Sequence Read Archive (SRA) database under BioProject PRJNA1077787. Metadata used and generated in this study are available at https://github.com/carlavieira1/Brisbane-arboviruses—metadata.
